# Peer Review of “Measuring Integrated Novel Dimensions in Neurodevelopmental and Stress-Related Mental Disorders (MIND-SET): Protocol for a Cross-sectional Comorbidity Study From a Research Domain Criteria Perspective”

**DOI:** 10.2196/36237

**Published:** 2022-03-29

**Authors:** Max V Birk

**Affiliations:** 1 Department of Industrial Design Eindhoven University of Technology Eindhoven Netherlands

**Keywords:** psychiatry, mental health, psychiatric disorders, neuropsychology, stress, comorbidity

*This is a peer-review report submitted for the paper “Measuring Integrated Novel Dimensions in Neurodevelopmental and Stress-Related Mental Disorders (MIND-SET): Protocol for a Cross-sectional Comorbidity Study From a Research Domain Criteria Perspective.”*

## Round 1 Review

Building on the Research Domain Criteria (RDoC; Cuthbert and Insel [1]), the manuscript [2] presents the study protocol of a transdiagnostic study program to determine mechanisms that either differentiate between neurodevelopmental and stress-related psychiatric disorders or show commonalities. The authors formulate a compelling argument that the pathophysiological pathway of psychiatric disorder needs to be considered taking a developmental perspective, with an emphasis on the role of comorbidities. To address such a high level of complexity, the authors present a cross-sectional study focused on stress-related (mood, anxiety, and substance abuse) and neurodevelopmental (autism, attention-deficit/hyperactivity disorder) disorders, with four points of measurements (distance unclear), and with each point of measurement including several observational levels: genetics, physiology, neuropsychology, system-level neuroimaging, behavior, self-report, and experimental neurocognitive paradigms.

Overall, I find this to be an extremely ambitious project. The study protocol as it is provides some good direction, and the approaches taken are state of the art, but the details of the proposal are inaccessible because of its complexity. What worries me most about the ambition of the plan is that the sample size and the requirements of the sample size are not discussed, which leads to issues with the interpretability of the collected data. An issue in a project that puts so much strain on the participants should be carefully considered.

I found the submission to be a mismatch to JMIRx Med; this is clearly a research protocol and might be better suited for JMIR Research Protocols.

Looking at the work solely from a research protocol perspective, I would like to read more details about how the authors intend to combine data or a detailed description of how they intend to pursue their analysis. The complexity prevents them from doing so, but as a result, the quality of the research protocol is difficult to judge—it is too high level to judge all aspects of the protocol responsibly. Defining the most relevant end points would be one approach that would help here.

Either way, I think the work is relevant to address, but journal fit and my mentioned points about sample and approach should be addressed, and the overall work would benefit from formatting and editing (some sections, for example, on the methods used, are redundant).

### Strengths

- Very important topic
- The authors pose a number of highly relevant questions
- Engaging summary of effects of individual disorders on pathophysiological and shared effect between disorders
- Considering the complexity of this project, the details are well thought through and the approaches described are reasonable. To assess the quality of each approach taken in detail, a range of expertise is required.

### Major Issues

- The sample size required is huge and one of the bottlenecks of the suggested approach; while the authors seem to have one unit to recruit participants, it is unclear how many participants would take part. The issue I foresee is that, with that many levels of observation, the complexity of comorbidities, and individual differences, the analysis will remain inconclusive. I would like to hear the authors’ thoughts on the sample size and interpretability of the collected data.
- The instruments used for data collection (questionnaires, biodata, etc) are all vaguely described (eg, which questionnaires will be used and, if biosamples are collected, what exactly will they be processed for). The data is provided in a later step—it is unclear to me why the same aspect is described twice with different levels of detail.
- Throughout the paper, it is not clear if the work has been performed, will be performed, or is still in the process of development and approval. This might be partially due to changes in time but also due to the overall presentation of the protocol—being more upfront about the goals of the manuscript would have helped.

### Minor Issues

- The formatting in the Word document and the PDF makes the document difficult to read. The Word document shows incorrect breaks and paragraphs, while the font in the PDF is pixelized.
- The citation format is not in line with JMIR standards.
- Acronyms like RDoC or MIND are not introduced at their first occurrence, which makes the interpretation difficult.
- Classifying autism as a disorder misses a neurodivergent perspective, which the autism community perceives, see [3].

## Round 2 Review

I want to thank the authors for such an in-depth, detailed, and carefully presented protocol. This is such a challenging study, but the presented implementation connects the different levels of inquiry and the patient groups very well. I found the decision made to be well motivated and am satisfied with the improvements.

I have one point that requires clarification:

– The authors aim to work with people diagnosed with autism spectrum disorder (ASD) but also included the command of language as an exclusion criterion (ie, “inadequate command of the Dutch language”). How will the authors make sure that not only vocal patients with ASD are included? From my understanding, selective mutism is quite common in people with ASD.

Several minor comments: overall, the manuscript requires proofreading and finishing touches.

### Abstract

“on the basis of” to “based on”

### Introduction

“the exception (1) .” to “(1).”

“on the basis of” to “based on”

### Current Approaches

“especially in light of” to “considering”

“Are depressive symptoms in someone with an autism spectrum disorder comparable to depressive symptoms in someone without an autism spectrum disorder?”; I assume that this should be attention-deficit/hyperactivity disorder in one of the cases.

“How well is someone with an autism spectrum disorder actually able to recognize and verbalize their mood symptoms, and how does this impact the diagnostic procedure, and the treatment choice and course?”; I suggest removing “actually”—it is unclear what the “actually” emphasizes, that there is little knowledge from a medical standpoint or if it emphasizes the assumption that people with autism are not aware of their own mood. I lack specialization in working with people with autism, but I would suggest to carefully frame neurotypical assumptions about neuroatypical processes.

### Comorbidity Within the RDoC Framework

“from a genetic, molecular or cellular level” to “from a genetic, molecular, or cellular level”

I stop commenting on this, but the use of the Oxford comma would help with readability when lists are used.

### Data-Driven Approaches

“has to be understood as step in” to “as a step towards”

### Study Aims and Outline

“mood, anxiety and substance abuse” to “mood, anxiety, and substance abuse”

### Methods

“are as well paid a small fee” — is there a reason the exact amount is omitted?

### Session 2: Behavioral Assessment

“faeces” to “feces”

“the Autism Spectrum Quotient ( AQ-50)” to “(AQ-50)”; “( NIDA)” to “(NIDA)”

“of the negative valence system”; unclear why underlined, maybe a subheading would differentiate the different systems discussed here better

### General Issues

Use of Oxford comma in lists

eg and ie should be followed by a comma. See [4].

Check the document for double spaces.

## Abbreviations

ASD: autism spectrum disorder
RDoC: Research Domain Criteria
